# The use of point-of-care ultrasound in Tshwane public and private sector emergency units

**DOI:** 10.4102/safp.v65i1.5711

**Published:** 2023-09-05

**Authors:** Nirvika Hurribunce, Vidya Lalloo, Benjamin V. Prozesky, Rulé Human, Detlef R. Prozesky, Maria M. Geyser, Andreas Engelbrecht

**Affiliations:** 1 Department of Family Medicine, Division of Emergency Medicine, Faculty of Health Sciences, University of Pretoria, Pretoria, South Africa; 2 Department of Family Medicine, Division of Emergency Medicine, Faculty of Health Sciences, Kalafong Provincial Tertiary Hospital, Pretoria, South Africa; 3 Division of Emergency Medicine, Faculty of Health Sciences, Steve Biko Academic Hospital, Pretoria, South Africa; 4 Department of Family Medicine, Faculty of Medicine, University of British Columbia, Kelowna, Canada; 5 Department of Medical Education, Faculty of Medicine, University of Botswana, Gaborone, Botswana

**Keywords:** point-of-care ultrasound, POCUS, emergency unit (EU), barriers to POCUS, indications for POCUS

## Abstract

**Background:**

The use of point-of-care ultrasound (POCUS) is an essential skill in the practice of emergency medicine (EM), with benefit to patient care by improving diagnostic accuracy. Despite this, there exists little data evaluating the use of POCUS in South African emergency units (EUs.).

**Methods:**

One hundred and seventeen doctors working in 12 public and private sector EUs in Tshwane were included. A questionnaire was used comprising of descriptive data regarding doctor demographics, levels of experience, and outcome data including POCUS frequency use, training level, indications for, and barriers to its use.

**Results:**

Many participants were general practitioners working in EUs (58.1%) followed by EM specialists and EM registrars. Of these participants, 88% used POCUS. Seventy one percent received informal POCUS training only. The indications for POCUS use were similar for both public and private sector, with no significant differences in overall use. The only significant association to POCUS use was age (> 33.3 years) and number of years since qualification (> 6.9 years.) Lack of and/or access to training were the main reasons for not using POCUS (18.8%.) There were no significant differences in the barriers to the use of POCUS between the sectors.

**Conclusion:**

Point-of-care ultrasound is used similarly in both public and private sector EUs in Tshwane. Lack of and/or access to POCUS training are the main barrier to its use.

**Contribution:**

This study underlines the state of POCUS use in Tshwane and highlights the barriers to its use, thus allowing academic heads and hospital managers to address them.

## Introduction

The utility of point-of-care ultrasound (POCUS) is expanding with new applications being described in the literature on a regular basis. It has been demonstrated that emergency physician performed POCUS improves diagnostic efficiency and accuracy, guides decision making and improves physician confidence.^1^ Point-of-care ultrasound use in conjunction with physical examination has been demonstrated in other African countries to clarify clinical cases by reducing the number of differential diagnoses.^2^ In resource-limited settings, POCUS can provide benefit to both patients and clinicians when formal radiological modalities are not readily available.^3^ Point-of-are ultrasound has also been demonstrated to be easy to teach in resource poor settings and that skills learned are maintained over time.^4,5^

The Canadian Association of Emergency Physicians recommends that emergency unit (EU) POCUS be available at all times.^6^ In the United States (US), the American College of Emergency Physicians includes POCUS training as a requirement for residents (registrars) currently training in emergency medicine (EM).^7,8^ Formal training with credentialing in POCUS is a requirement for registrars training to be EM specialists in South Africa. As EM is a relatively new and evolving speciality in South Africa, many EUs are staffed by general practitioners or medical officers.^9^ Accredited POCUS training programmes are open to specialists and non-specialists in South Africa and most often consist of modules including extended focused assessment by sonography in trauma (eFAST), central and peripheral vascular access, focused emergency echocardiography in resuscitation (FEER), deep venous thrombosis (DVT), abdominal aortic aneurysm (AAA) assessments and lung ultrasound.^10^

The focused assessment by sonography in trauma (FAST) examination has been shown to have a sensitivity of 90% and specificity of 99% – 100% for the presence of intraperitoneal free fluid in blunt and penetrating abdominal trauma.^8,11^ Patients receiving FAST exams have been found to accelerate access to the operating room, require fewer computed tomography (CT) scans, have shorter hospital stays and experience fewer complications than those not undergoing FAST.^12^ Data are also available that show patient benefit when POCUS is performed with suspected ectopic pregnancy,^8,13,14^ suspected AAA,^8,15,16^ emergent echocardiography and haemodynamic assessment,^8,17,18^ suspected hepatobiliary disease,^8,19^ suspected DVT,^8,20^ suspected pneumothorax^8,21^ and for procedural guidance.^8,22^

In research performed abroad (specifically in Quebec^7^ and Ontario,^23^ Canada,) varying proportions of EU physicians were found to be using POCUS for a range of indications similar to those taught in South African POCUS training modules. Surveys performed in Canada (in Quebec,^7^ Ontario^23^ and Newfoundland^24^) as well as the Washington, Wyoming, Alaska, Montana and Idaho (WWAMI) region of the US^25^ consistently found that a lack of equipment, training, funding and difficulty maintaining skills were the dominant barriers to the use of EU POCUS.

Closer to home, there has been an emphasis on the implementation and maintenance of POCUS training in Africa,^26^ and inadequacies in the current South African POCUS curriculum for EM specialist trainees have been explored.^10,27^ However, research that assesses the actual degree to which POCUS is used by doctors (of all levels of expertise) in South African EUs is lacking. Numerous studies have demonstrated that patients benefit from POCUS in EUs and POCUS appears to be relatively easy to teach. However, no data are available to illustrate how many South African doctors (in both the public and private sectors) are using POCUS, the indications for which they use it, and the potential barriers to its use.

The provision of healthcare in South Africa has a two-tiered, and highly unequal system. The public sector is state-funded and serves a larger proportion (71%) of the population, with restricted resources.^9^ The private sector is largely funded through individual contributions to medical aid schemes or health insurance. This discrepancy in the provision of healthcare in terms of both human and physical resources could be expected to impact the use of POCUS across the two sectors.

The objective of this study was to determine the proportion of doctors who use POCUS in public and private EUs in and around the City of Tshwane Metropolitan Municipality, South Africa. The study questionnaire included demographic data on the EU doctors, the frequency of POCUS use, barriers to and factors that aid the use of POCUS. A secondary aim was to establish whether doctors in private sector versus public sector EUs use POCUS to different degrees.

## Research methods and design

A cross-sectional study design was used. Data were collected using an adapted questionnaire that had been designed and used for the purpose of collecting data in a similar Canadian study,^7^ which assessed the use of EU POCUS in rural Canada. Permission was obtained from the Canadian authors. In compliance with survey design recommendations, the questionnaire was pre-tested on a statistically sound convenience sample of 15 EU doctors (to ensure clarity and relevance) and was adapted based on the result.

The questionnaire included 24 questions comprising quantitative and qualitative data. Descriptive data included doctor demographics (age, gender, qualifications, experience), average number of EU shifts per year, number of patients seen per year at the EU in which employed, access to CT scan and radiology services, access to portable ultrasound machine and whether employed at a public or private sector hospital. Outcome data included information on the use of POCUS (use, indications for use, frequency of use), level and type of POCUS training and experience, as well as aids and barriers to the use of POCUS.

This questionnaire was administered to doctors working in 12 hospitals in the City of Tshwane Metropolitan Municipality between 2020 and 2022. Of these EUs, eight were part of the public sector and four were private sector hospitals. This discrepancy was because of some private hospitals withholding permission to conduct research during and after the coronavirus disease 2019 (COVID-19) pandemic. The EUs included provide a range of care from primary-level care to district hospital care and tertiary-level care. Sampling consisted of a convenience sample of EU physicians who were on duty when the investigator visited the EU. In instances where physical visits to EUs by the investigator were not deemed feasible, electronic questionnaires were distributed using a secure online tool (SurveyMonkey) and were completed by the doctors in digital form. This form of data collection became relevant when restrictions on EU visits by the investigator arose during the COVID-19 pandemic.

Any doctor who provided patient care in the EU was regarded as an EU physician: regardless of qualifications, training, or EU experience. These physicians were categorised as follows: community service medical officers were defined as doctors who were registered as such with the Health Professionals Council of South Africa (HPCSA) and were currently in the year following their supervised internship training. Medical officers and general practitioners were defined as non-specialist doctors who were registered as independent practitioners with the HPCSA. Registrars were defined as trainee-specialists with HPCSA classification. Lastly, specialists were defined as the doctors who held HPCSA specialist registration. Medical interns were excluded.

A total of 117 questionnaires were completed and included in this study. A sample of 97 EU doctors was deemed appropriate to provide a reliable estimate of the proportion of interest with 95% confidence. However, doctors within a facility may not have had entirely independent practice patterns and opinions. In order to compensate for design effect, it was subsequently calculated that a sample size of at least 117 doctors was required.

Data analysis was performed under the survey (svy) command in Strata^®^ release 15 making use of sampling weights. The study applied descriptive statistics and the Chi-square test to test the association between outcome and hospital type. All testing was performed at a 0.05 level of significance.

### Ethical considerations

Ethical approval for this study was granted by the Faculty of Health Science Research Ethics committee of the University of Pretoria (Ethics Reference no.: 496/2019). Informed consent was attained from the hospital CEO and/or manager of the respective EUs, as well as the doctors who participated in the study. Questionnaires were completed anonymously thereby ensuring the confidentiality of the participants and adhering to the *POPI Act*.

## Results

### Demographics

There were 117 participants, of which 76% were female and 24% were male (Table 1).

**TABLE 1:** Demographic data comparing the use of ultrasound in public and private sector emergency units.

Descriptive variable	Description	Private		Public		*p*
		*n*	%	*n*	%	
Gender	Female	19	76	70	76	0.7089
	Male	6	24	22	24	
Total shifts in the EU per year	< 40	4	16	6	7	0.0874
	41–80	1	4	17	18	
	81–120	0		10	11	
	121–160	6	24	8	9	
	> 160	14	56	51	55	
Total number of patients seen in the EU per year	Unknown	2	8	3	3	0.8350
	< 5000	4	16	16	17	
	5000–9999	3	12	9	10	
	10 000–14 999	3	12	5	5	
	15 000–19 999	1	4	8	9	
	> 20 000	12	48	51	55	
Access to CT scanner in hospital	No	1	4	8	9	0.0410
	Yes, 24/7	21	84	81	88	
	Yes, sometimes	3	12	3	3	
Availability of radiologist-performed ultrasound in hospital	No	0		5	5	0.5924
	Yes, 24/7	12	48	53	58	
	Yes, sometimes	13	52	34	37	
Portable ultrasound machine in EU	No	4	16	4	4	0.0795
	Yes	21	84	88	96	

CT, computed tomography; EU, emergency unit.

**FIGURE 1:**
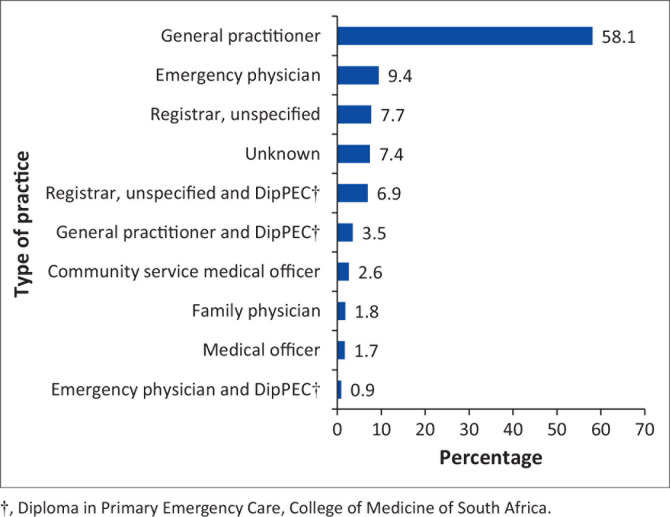
Classification of study participants based on type of practice.

Participants from private hospitals had older participants (mean age of 34.32 years) compared with 32.29 years in the public hospital participants. The majority of participants classified themselves as general practitioners (58.1%) followed by EM specialists (8.5%) and registrars (7.7%) (Figure 1). Most participants were employed in public hospitals (79%) compared with participants employed in private hospitals (21%). More than half of the participants (55.6%) worked more than 160 EU shifts per year, with the majority of participants (53.8%) stating that more than 20 000 patients are seen per year in their EUs. In terms of level of experience, the mean time since qualification in the private hospital participants was 87 months compared with 80 months in the public hospital participants. Emergency medicine experience was noticed to be higher in the private hospital participants (mean of 78 months) compared with public hospital participants (mean of 49.1 months).

With regard to training, results indicated that 70.9% of participants reported having received only informal POCUS training. More than half of these participants (55%) received the informal training from EM specialists who are HPCSA-accredited POCUS instructors, followed by 28% who received training from EM specialists who are not credited as instructors. Formal ultrasound training was completed by only 37.6% of participants, and of these, 36.8% completed the Emergency Medicine Society of South Africa (EMSSA) Basic Emergency Ultrasound Level 1 course and 13.7% had completed the EMSSA Advanced Level 2 Ultrasound course. The Echo-guided Life Support (EGLS) course was completed by 20.5% of these participants. Further accredited courses completed by participants were provided by critical care training providers and providers who trained only in eFAST. Of the participants who completed the formal ultrasound courses, only 18.8% of them completed credentialling processes to hold formal ultrasound certification. Overall, most participants (74.4%) reported plans to complete further training in POCUS.

### Access to radiological services

Results showed that 87.2% of participants had access to CT scans at all times in their hospital. A large proportion of participants (55.6%) also had access to radiologist-performed ultrasound in their hospital compared with 40.2% who only have this resource sometimes. The majority of participants also had access to a portable ultrasound machine in their EU (93%). There were no statistically significant differences between the public and private sector hospitals except for access to CT scan (Table 1).

### Use of point-of-care ultrasound

**FIGURE 2:**
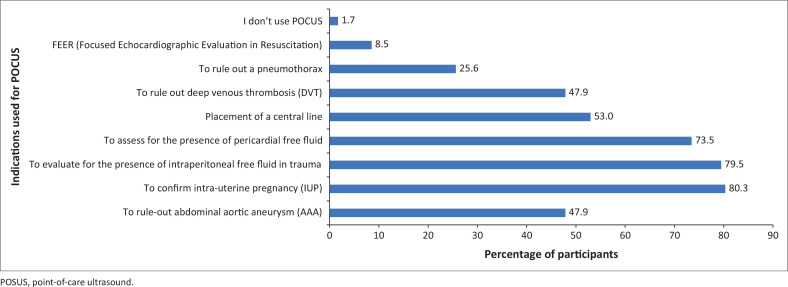
Indications for the use of point-of-care ultrasound by the study participants.

Most participants (82.1%) strongly agreed that POCUS is a skill at which an emergency physician should be competent. Furthermore, 74.4% of participants strongly agreed that POCUS is an essential skill for an emergency physician in a resource poor setting. A total of 88% of participants reported using POCUS in their practice with 47.9% of participants often using POCUS more than once per shift. A total of 29% of participants used POCUS less than once per week. The indications for using POCUS were mainly to confirm intra-uterine pregnancy (80.3%); to evaluate for the presence of intraperitoneal free fluid in trauma (79.5%) and to assess for the presence of pericardial free fluid (73.5%), as shown in Figure 2.

### Barriers to the use of point-of-care ultrasound

A lack of training was indicated as the main reason for not using POCUS (18.8%) followed by fear of medico-legal problems (6.8%). One third of those participants who cited a lack of training as the reason for not using POCUS reported difficulty with accessing POCUS courses that were often over-subscribed. The most common reported barriers in the qualitative data were a lack of training and an adequate ultrasound machine or broken ultrasound machines. Other noticed variables included a lack of experience or availability of supervision and a workload and/or volume of patients that was too high to be able to perform ultrasound adequately. It is notable that 90% of participants who reported no barriers to training worked in EUs where an EM specialist and/or HPCSA-accredited POCUS instructor was present.

### Comparison of the use of point-of-care ultrasound in public and private sector emergency units

In terms of training, most participants in both sectors had informal training, yet no formal training and no formal credentialing or certification in the use of POCUS. The study demonstrated that training in POCUS had a significant impact on POCUS usage (odds ratio [OR]: 31.62; 95% CI: 5.55;169.8; *p* < 0.001). The course completed by most participants who had formal training in both sectors was the EMSSA basic emergency ultrasound level 1 course. The majority of participants in both sectors agreed that POCUS is a skill with which an emergency physician should be competent and that POCUS is an essential skill for an emergency physician in a resource-poor setting. However, no significant association was demonstrated between these results by the hospital sector.

The majority of participants in both private and public hospitals used POCUS in practice (76% and 91%, respectively), with no statistically significant association between these results. There was a statistically significant difference (*p*-value < 0.005) noticed in the frequency with which participants used POCUS between private and public EUs. The majority of participants in private EUs (52%) used POCUS less than once per week while the majority of those in public EUs used POCUS more than once per shift (55%).

Participants younger than the mean age (33.3 years) and those with less years of experience (less than 6.9 years) were less likely to use POCUS. The use of POCUS was not significantly associated with any hospital sector.

In public EUs, most participants used POCUS to evaluate for the presence of intraperitoneal free fluid in trauma (85%), followed by the confirmation of intrauterine pregnancy (83%) and the assessment of the presence of pericardial free fluid (77%). In private EUs, results showed that most participants used POCUS to confirm intrauterine pregnancy (70%), followed by use for placement of central lines (64%). Point-of-care ultrasound was further used to evaluate for the presence of intraperitoneal free fluid in trauma (60%) and to assess for the presence of pericardial free fluid (60%). There was no significant difference between participants from private and public EUs concerning the indications for which POCUS was used.

The most common barrier to POCUS use in both public and private sector hospitals was the lack of training. Private sector participants had more difficulty in accessing POCUS courses (as they were reported as often full) while public sector participants indicated that accessing training was not problematic. There was no significant association and difference between the sectors demonstrated.

## Discussion

Our study found that the majority of EU physicians in Tshwane classified themselves as general practitioners and most (both in public and private sectors) used POCUS in their practice. This was despite the majority of EU physicians having had only informal training in the use of POCUS. Informal training for most participants who used POCUS was provided by EM specialists who are also HPCSA-accredited POCUS instructors. The most common indications for POCUS use were to confirm intra-uterine pregnancy, to evaluate for the presence of intraperitoneal free fluid in trauma and to assess for the presence of pericardial free fluid. Significant factors that increased the use of POCUS were the number of years since qualification and the history of being trained in POCUS. The most common barrier to POCUS use was the lack of training in POCUS, with availability of POCUS courses frequently identified as a problem. It was also noticed that the only significant difference in the use of POCUS between EUs in the public and private sector hospitals was the frequency of use, with POCUS used more frequently in the public sector EUs. This is not surprising as the majority of South Africa’s population have access to public hospitals only.

In research performed abroad in rural centres in Quebec, Canada, Léger et al.^7^ found that most EU physicians had access to a dedicated ultrasound service and most used POCUS. The POCUS was generally used for ruling out AAAs, ruling in intrauterine pregnancy, ruling out intraperitoneal free fluid and ruling out pericardial effusion. The top four indications in our study population were similar. However, this study did not include AAA assessments, but included ultrasound guided central line insertion. Another similarity was that EU POCUS use was found to be widespread in Quebec and that access to training was a significant barrier to the use of EU POCUS. This may reflect a similar lag between real-world practice and undergraduate medical education.

In contrast, the Ontario study^23^ (which was performed in rural hospitals) found that few EU physicians had access to an ultrasound machine and less than half knew how to perform ultrasonography in any form. A lack of machine availability was not found to be a major barrier in our study (16% in the public sector and 4% in the private sector did not have access to an ultrasound machine). The authors went on to state that this constituted a gap in care that needed to be addressed. Our study was conducted in the Tshwane Metropolitan area, which is considered an urban setting, and this may explain the differences in barriers found.

In terms of demographics, in the Quebec study^7^ the participants had a median age of 37 years and a median of 7 years of practice, with 93% having identified themselves as family physicians certified by the College of Family Physicians of Canada. The median age of the EU physicians included in the Ontario study^23^ was 49 years, with the participants having been in practice for a median of 20 years. This is in contrast to our study where the EU physicians were younger (mean age of 33.3 years) and less experienced (mean of 6.9 years of practice.) The younger, less experienced participants in the Quebec study^7^ were similar in demographics to participants in our study. However, a much smaller proportion of our participants were specialists in their field (majority identified as general practitioners). Despite this fact, the use of POCUS overall was similar between our study and the Quebec study.^7^ The number of specialists versus general practitioners was not described in the Ontario study.

A study^25^ conducted in the WWAMI region of the US found that a lack of equipment, training, funding and difficulty maintaining skills were the dominant barriers to the use of EU POCUS. In comparison, although a lack of equipment and broken equipment were cited as barriers in the current study, the predominant barrier was a lack of training because courses being fully booked. This reflects apparently good access to ultrasound equipment in the sampled Tshwane metropolitan hospitals. In this regard, it is interesting that an upper-middle-income country may be better equipped with ultrasound equipment than a high-income country.

In Africa, a study^28^ conducted in an urban tertiary hospital in Addis Ababa, Ethiopia, showed that the main indications for EU POCUS were for the assessment of trauma, medical shock and undifferentiated dyspnoea. A similar study^29^ conducted in the largest national referral hospital in Tanzania evaluated their most common indications for EU POCUS to be trauma, respiratory presentations and abdomino-pelvic pain. It is notable that in the current study, only ruling out a pneumothorax was identified as an indication for ultrasound related to respiratory presentations and was only used by 25.6% of participants. Similar to these two studies, trauma was one of the most common indications for EU POCUS (79%) in our study. It is surprising that the participants of these two African studies did not highlight the use of POCUS to rule in intra-uterine pregnancies, as was the case in our study.

Regarding barriers to POCUS use, a survey^30^ was administered to clinical educators deployed to academic hospitals in Tanzania, Malawi and Uganda as part of the Global Health Service Partnership (an initiative from the US providing healthcare human resources and training to resource-limited countries). These clinical educators cited the largest perceived barrier to EU POCUS as a lack of ultrasound knowledge. This is in line with the main barrier to POCUS use shown in our study.

The current study provided insights into the current use of POCUS in EUs in Tshwane. This information may be used by stakeholders such as universities and ultrasound teaching institutions to decide on teaching curricula. It may also be helpful to health institutions that wish to introduce POCUS, to become aware of the main barriers to the use of POCUS.

There were notably more responses from EU physicians in the public-sector hospitals compared with private-sector hospitals. More responses from the private sector could improve the analysis of the results and provide a better insight into the potential differences between the sectors. Smaller rural hospitals were not sampled so these results are not generalisable to this population. Despite the fact that a small number of EM physicians were surveyed (which should be seen in the context regarding access to physicians and hospitals during the COVID-19 pandemic) the findings of this study remain highly relevant and important. As this was a survey, data were only collected at a snapshot in time and within a defined area. This study was conducted through convenience sampling, which may not be the most reliable representation of the study population.

Our study revealed that the most important barrier to POCUS use was a lack of and/or access to training. As such, we make the following recommendations:

- Curriculum committees of universities should include POCUS training modules into the undergraduate medical curricula.
- The HPCSA should consider POCUS training and/or credentialing as a requirement to complete medical internship training.
- Widen access to POCUS training through online courses and centralised ultrasound facilities at public teaching hospitals and in the private sector.
- Emergency unit managers should develop a training plan for less-experienced doctors, which may include emphasis on more doctors becoming accredited POCUS instructors.

This study provides an ideal platform to commission a more extensive and comprehensive assessment of the need for, and use of, POCUS at a national level, which should include public and private healthcare institutions in urban and rural settings.

## Conclusion

The use of POCUS in EUs in Tshwane is similar between the public and private sectors. Access to POCUS training and its use in public and private sector emergency units is a major barrier. The major indications for the use of POCUS were the ruling in of intra-uterine pregnancies and eFAST in trauma patients.
